# Regulation of chromatin structure by poly(ADP-ribosyl)ation

**DOI:** 10.3389/fgene.2012.00169

**Published:** 2012-09-03

**Authors:** Sascha Beneke

**Affiliations:** Institute of Veterinary Pharmacology and Toxicology, University of ZurichZurich, Switzerland

**Keywords:** poly(ADP-ribosyl)ation, PARP1, chromatin, recruitment, transcription, CTCF

## Abstract

The interaction of DNA with proteins in the context of chromatin has to be tightly regulated to achieve so different tasks as packaging, transcription, replication and repair. The very rapid and transient post-translational modification of proteins by poly(ADP-ribose) has been shown to take part in all four. Originally identified as immediate cellular answer to a variety of genotoxic stresses, already early data indicated the ability of this highly charged nucleic acid-like polymer to modulate nucleosome structure, the basic unit of chromatin. At the same time the enzyme responsible for synthesizing poly(ADP-ribose), the zinc-finger protein poly(ADP-ribose) polymerase-1 (PARP1), was shown to control transcription initiation as basic factor TFIIC within the RNA-polymerase II machinery. Later research focused more on PARP-mediated regulation of DNA repair and cell death, but in the last few years, transcription as well as chromatin modulation has re-appeared on the scene. This review will discuss the impact of PARP1 on transcription and transcription factors, its implication in chromatin remodeling for DNA repair and probably also replication, and its role in controlling epigenetic events such as DNA methylation and the functionality of the insulator protein CCCTC-binding factor.

## Poly(ADP-ribosyl)ation

Poly(ADP-ribosyl)ation as enzymatic reaction is known since the early sixties of the last century (Chambon et al., 1963). In the following 20 years it was related to several nuclear functions, i.e., histone modification (Aubin et al., 1982), differentiation (Farzaneh et al., 1982; Pekala and Moss, 1983), cell death (Sims et al., 1983), transcriptional regulation (Slattery et al., 1983) and DNA repair/genome stability (Davies et al., 1978; Durkacz et al., 1980). Also the major players were analyzed:

1. Structure of the product poly(ADP-ribose) (PAR) (Chambon et al., 1966; Nishizuka et al., 1967; Reeder et al., 1967),
2. Synthesizing enzyme poly(ADP-ribose) polymerase(−1) (PARP1) [(Tsopanakis et al., 1976), cDNA cloned in (Cherney et al., 1987; Suzuki et al., 1987)] and
3. Degrading enzyme poly(ADP-ribose) glycohydrolase (PARG) [(Ueda et al., 1972), cDNA cloned in (Lin et al., 1997)].

In the enzymatic reaction NAD^+^ is cleaved into nicotinamide and ADP-ribose, with the latter attached to glutamate or aspartate via an ester bond (Ogata et al., 1980b), and to lysine, forming a ketoamine by Schiff-Base and Amadori rearrangement (Altmeyer et al., 2009). Whereas esters are enzymatically easy to revert, ketoamines show substantial stability and may form a “modification-mark” on the respective protein. After attachment of the first ADP-ribose moiety, further units are rapidly added via α-gylcosidic bonds and branches can originate from the growing chain, depending on the synthesizing enzyme and interaction partner (Naegeli and Althaus, 1991).

PARPs are nowadays a family of 17 enzymes, but not all of them are active ADP-ribose transferases and only few show truly polymerizing activity (Hottiger et al., 2010). In case of PARP1, the product poly(ADP-ribose) displays a tree-like structure, forming a highly negative charged cloud at the covalently modified protein, which impacts on functionality probably through electrostatic repulsion of affected enzymes from DNA (Zahradka and Ebisuzaki, 1982). The main acceptor of PAR is PARP1 itself (Ogata et al., 1981), but also its interaction partners can be modified, as shown for several nuclear proteins *in vitro* and *in vivo*. Degradation of the polymer is performed by PARG in an endo- as well as exoglycosidic reaction, releasing PAR of different length as well as ADP-ribose monomers (Meyer-Ficca et al., 2004; Bonicalzi et al., 2005). Enzymatic activity of PARP1 is very low and PAR in unstimulated cells has an estimated half-life of up to several hours (Alvarez-Gonzalez and Althaus, 1989). After application of DNA strand-break inducing agents, PARP1 dimerizes at the break, leading to its activation (Mendoza-Alvarez and Alvarez-Gonzalez, 1993; Jorgensen et al., 2009; Langelier et al., 2012). PARP1 can also bind non-B-DNA structures (Soldatenkov et al., 2002; Lonskaya et al., 2005; Potaman et al., 2005). PAR synthesized in this process displays a much reduced half-life of less than a minute as high local concentrations of the polymer stimulate PARG activity (Alvarez-Gonzalez and Althaus, 1989).

Increased poly(ADP-ribosyl)ation (PARylation) metabolism is one of the first cellular responses following exposure to genotoxic stress (Haince et al., 2007, 2008). In addition to covalent modification proteins can interact with PAR in a non-covalent fashion. So far, three different motifs have been described:

First, a sequence of basic and hydrophobic residues, the so called PAR-Binding-Motif (PBM) (Pleschke et al., 2000), which is present in many proteins involved in maintaining genomic stability, i.e., telomerase, p53, histones, base-excision-repair (BER) platform protein XRCC1, nucleotide-excision-repair (NER) protein XPA and many more.

Next, it was reported that the macro-domain binds in an end-capping mode to the tip of a PAR chain (Karras et al., 2005).

Finally, a PAR-Binding-Zinc finger (PBZ) was discovered in APLF, a histone chaperone (Ahel et al., 2008).

The wide-spread regulatory impact of PARylation has been described in a recent publication (Gagne et al., 2012). A large scale analysis of PAR-interacting proteins after application of genotoxic stress revealed that specific proteins are associated with PAR in a sequential way after challenge, with an early group representing repair complexes, followed by translation regulators and finally factors involved in RNA processing. Both principles, covalent and non-covalent interaction, can be present side-by-side within one protein. For example the tumor suppressor p53 displays three covalent as well as three non-covalent binding sites (Fahrer et al., 2007; Kanai et al., 2007). Interestingly, the interaction partner is one determinant that affects complexity of PAR, i.e., chain-length and branching (Naegeli and Althaus, 1991). Additionally, proteins differ in their ability to bind to different PAR structures (Fahrer et al., 2007).

In summary, PARP1 (respectively its product PAR) is able to change the surrounding environment by either excluding modified proteins from distinct sites, or by attracting factors containing PAR interaction-motifs.

## PARP1 in DNA-repair and replication

### Single-strand break repair and histone shuttle

Activity of PARP1 has been correlated with DNA damage since it was discovered (Miller, 1975a,b). DNA strand-breaks are strong inducers of PARylation, stimulating the enzyme several hundredfold. The exact cellular function of this energetic costly reaction was long unclear, but application of genotoxic agents with simultaneous suppression of PARylation led to increased persistence of breaks (Morgan and Cleaver, 1983), reduced repair (Yamamoto and Okamoto, 1982) and enhanced sister-chromatid-exchanges (Hori, 1981; Otsuka et al., 1983; Park et al., 1983; Meyer et al., 2000), indicating that PARP1 activity is intimately involved in maintaining genomic stability. As histones have been reported early as covalent acceptors of PAR (Aubin et al., 1982), disassembly of nucleosomes to facilitate repair was suggested. Soon after this theory, *in vitro* experiments showed that purified PAR added to polynucleosomes was able to relax their condensed structure (Poirier et al., 1982). This pointed to non-covalent interaction between at least the linker histone H1 and PAR. Indeed, affinity of H1 to polymer is strong enough to resist phenol partitioning (Panzeter et al., 1992). In addition, also core histones have been shown to be covalently (Ueda et al., 1975; Ogata et al., 1980a; Messner et al., 2010) and non-covalently (Adamietz and Rudolph, 1984; Kreimeyer et al., 1984) modified.

These data led to the assumption that one of the major tasks of PAR synthesis is to clear DNA from nucleosomes by direct modification as well as binding of histones to polymer, granting access of repair factors to the lesion (Mathis and Althaus, 1987; Realini and Althaus, 1992). The detection of PBMs in histones and many other proteins related to DNA repair and stress response, i.e., tumor suppressor p53, cyclin-dependent kinase inhibitor p21, base-excision- and single-strand break-repair protein XRCC1, nucleotide-excision repair protein XPA, DNA-Pol Σ, telomerase subunit TERT, Ku70 and mismatch-repair protein MSH6 (Pleschke et al., 2000), corroborated the hypothesis of PARP1 as a repair and cell cycle regulator. This was confirmed *in vivo* by the fact that the BER adaptor protein XRCC1 (X-ray repair cross-complementing protein 1) depends on PAR for its recruitment to lesions. Inhibition or knockout of PARP1 strongly impacts on XRCC1 enrichment at DNA strand breaks (El-Khamisy et al., 2003). XRCC1 interacts as shuttle with proteins necessary to perform the synthesis and resealing steps after incision as DNA Polβ, polynucleotide kinase and DNA ligase III. Direct interaction of PARP1 with DNA ligase III may help in formation and guiding of the productive complex ((Leppard et al., 2003).

**Figure 1:**
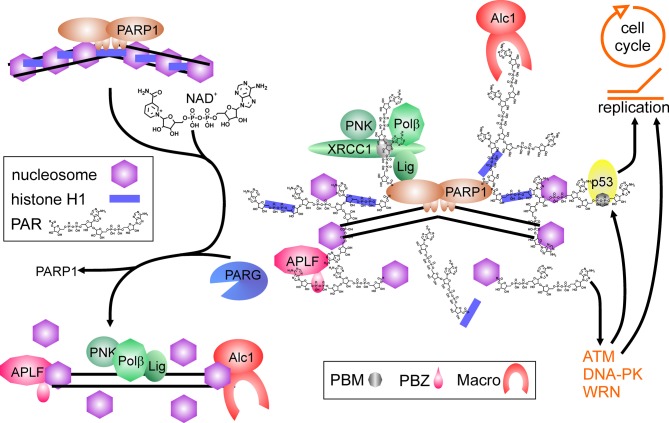
**Poly(ADP-ribose) polymerase1 in DNA repair.** Binding to DNA breaks and dimerization activates PARP1, which synthesizes poly(ADP-ribose) (PAR) from NAD^+^, covalently modifying itself and neighboring proteins, i.e., histones (blue bars = histone H1, purple hexagons = core histones). Proteins containing PAR interaction motifs (PBM = PAR binding motif, PBZ = PAR binding zinc finger; Macro = macro-domain) are recruited to the site of damage, whereas histones in the vicinity are displaced from DNA. Auto-modification of PARP1 abrogates PARP1 DNA binding. Recruited proteins like the XRCC1-complex containing PNK (polynucleotide kinase), Polβ (DNA polymerase β) and Lig (DNA ligase III) are released from PAR chains by degrading activity of PARG and can perform repair on the nucleosome-free DNA. Histone chaperones as APLF and Alc1 may help in disassembling and reassembling of histones on DNA. Binding of PAR to p53 (either covalent or non-covalent) as well as interaction of PARP1 and PAR with proteins like ATM, DNA-PK and WRN regulate cell cycle progression and replication.

Thus, PARP1 and its activity are important regulators of DNA nick-repair. Shortage of the substrate NAD^+^ or strong activation may limit efficiency of repair, as PARP1 binds tightly to DNA breaks if no auto-modification takes place (Satoh and Lindahl, 1992; Satoh et al., 1994), and hyperactivation may shift the spectrum of PARP1 protein-substrates. This is in line with studies showing increased genomic instability by application of PARP inhibitors, and at least *in vitro*, PARP1 is able to inhibit DNA polymerases α and β as well as DNA ligase II by covalent modification (Yoshihara et al., 1985). This could represent a regulatory mechanism to avoid futile repair attempts of cells suffering from a high burden of DNA damage. PARP1 also interacts and stimulates flap-endonuclease-1 (FEN1), responsible for cleaving exposed DNA single strands (flaps) derived from strand-displacement synthesis during BER or replication (Prasad et al., 2001). Finally, the chromatin remodeler Alc1 (Ahel et al., 2009; Gottschalk et al., 2009) and APLF1, a histone chaperone including AP-endonuclease activity (Eustermann et al., 2010; Mehrotra et al., 2011), are recruited and activated upon PAR binding, probably facilitating nucleosome disassembly and re-assembly before and after repair process (Figure 1).

### Double-strand break repair and replication

PARP1 also regulates signaling in double strand break repair (DSBR). Inhibition of PARylation hampers and delays activation of initiator PI3K-related kinase ATM (ataxia telangiectasia mutated) (Haince et al., 2007), and ATM forms a complex with PARP1 (Aguilar-Quesada et al., 2007). There is evidence that also DNA-PK directly interacts with and is stimulated by PARP1 (Ruscetti et al., 1998). The interaction of DNA-PK and PARP1 is strengthened by the observation that suppression of the activity of one of them negatively affects the functionality of the other *in vitro* (Veuger et al., 2004). In addition to these two important damage-signaling kinases, PARP1 has many overlapping interaction partners with WRN, a RecQ helicase with exonuclease activity mutated in the Werner adult premature aging syndrome. WRN is responsible for resolving DNA structures such as Holliday junctions and repair intermediates. It participates in BER, DSBR, replication and maintenance of telomeres, the latter one by proper opening the protective *t*-loop. WRN and PARP1 directly interact and regulate each other (Adelfalk et al., 2003; von Kobbe et al., 2003, 2004), and are able to form a complex with the DNA-PK subunits K70/Ku80 (Li et al., 2004). In this regard, it is interesting to note that FEN1 also interacts with WRN in BER and at telomeres (Brosh et al., 2001; Sharma et al., 2003), where also PARP1 activity is needed to maintain proper length (Beneke et al., 2008). Another cellular site were all three proteins—FEN1, WRN, and PARP1—are located together is the replication complex (Sharma et al., 2004). It has been shown that PARP1 modifies at least 15 different proteins in the complex, most prominently DNA Polα, topoisomerase I (TopoI) and proliferating cell nuclear antigen (PCNA), but it is unclear if PARylation is needed for proper assembly of replication complex or for regulation of its functionality (Simbulan-Rosenthal et al., 1998). Poisoning of TopoI stalls replication forks, and reversal of this depends on PARP1 activity (Ray Chaudhuri et al., 2012), probably by reactivating TopoI and induction of repair (Malanga and Althaus, 2004).

## PARP1 in transcription

### PARP1 activity as negative controller of transcription

**Figure 2:**
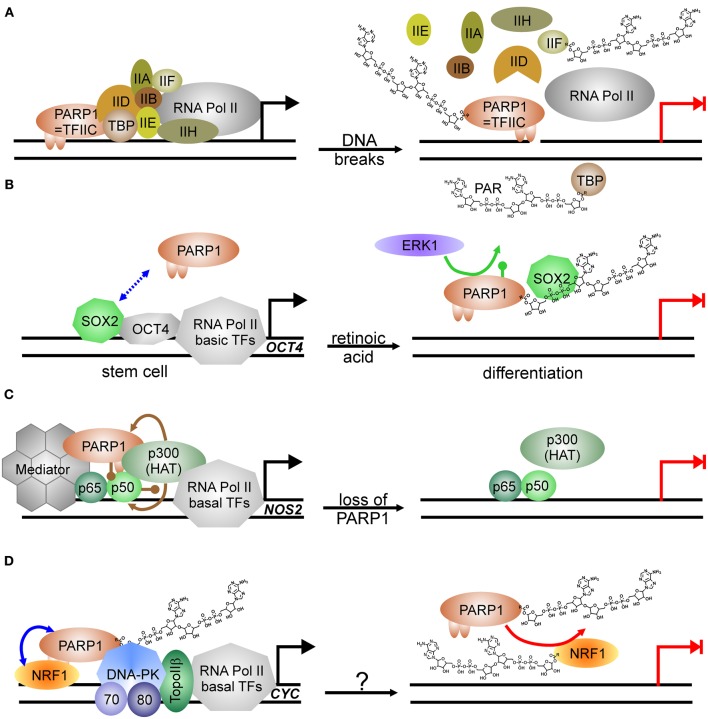
**PARP1-activity mediated suppression of transcription. (A)** PARP1 as basal transcription factor TFIIC monitors DNA breaks in the vicinity of promoters. Transcription machinery is disassembled at pre-initiation complex formation due to modification of TBP (TATA-binding protein) and TFIIF with PAR after DNA damage induction. Transcription is blocked (switch from black arrow to blocked red arrow). **(B)** PARP1 in regulation of stem cell differentiation. SOX2 weakly interacts with PARP1 (dashed double-headed blue arrow). Phosphorylation (green lollypop) of PARP1 by kinase ERK1 leads to auto-modification of PARP1. SOX2 DNA-binding and dimerization with OCT4 is disrupted by interaction with PARylated PARP1. Transcription is abrogated (switch from black arrow to blocked red arrow). **(C)** Positive impact of PARP1 protein itself on transcription as co-activator of NFκB. At the *NOS2* promoter, PARP1 is acetylated (brown lollypops) by p300 HAT (histone acetyl-transferase), which also acetylates NFκB, and interacts thereafter with NFκB subunit p50. Binding of co-activator Mediator to the complex is stabilized by PARP1 and facilitates transcription. Loss of PARP1 and also putatively its activation disrupts transcription complex. Transcription is abrogated (switch from black arrow to blocked red arrow). **(D)** PARP1 as co-activator and PARP1 activity as repressor. PARP1 complexes with NRF1 irrespectively of its own modification status (blue double-headed arrow). Covalent modification of NRF1 with PAR (red arrow) disrupts the permissive transcription complex containing DNA-PKcs/Ku70/Ku80 and TopoIIβ, releasing NRF1 from DNA. Transcription is blocked (switch from black arrow to blocked red arrow). The respective stimulus needs to be determined (question mark).

Transcription by RNA Pol II is regulated in multiple ways, i.e., by induced assembly of different specific transcription factor complexes at susceptible promoters. In addition, general transcription factors—named TFII followed by a letter—are needed for proper transcription of any gene [see Thomas and Chiang (2006) for review]. PARP1 has been isolated in 1983 as TFIIC, necessary for suppression of transcription initiation at nicked DNA (Slattery et al., 1983). Activated PARP1 abrogates formation of the pre-initiation complex (PIC) (Oei et al., 1998b) by PARylating the TATA-binding protein (TBP) (Oei et al., 1998a) and TFIIF (Rawling and Alvarez-Gonzalez, 1997) (Figure 2A). Similarly, specific transcription factors as YY1, p53, CREB, Sp1, and NFκB are prevented from binding to their respective recognition sequence if PARylated (Wesierska-Gadek et al., 1996; Oei et al., 1997; Chang and Alvarez-Gonzalez, 2001; Mendoza-Alvarez and Alvarez-Gonzalez, 2001). PARylation negatively controls also the function of transcription factors essential in sex-determination via SRY, and maintenance of “stem-ness” of cells via SOX2. SRY (sex-determining region of Y) is the master regulator in sex-determination and essential for testis development. SRY-mediated transcription is severely impaired upon PARP1 stimulation, as its covalent modification abrogates interaction with its cognate DNA-binding sequence (Li et al., 2006). SOX2 acts in concert with OCT4 in stem-cell maintenance. Both form a complex on respective promoters/enhancers, i.e., *NANOG* and *SOX2* and *OCT4*, leading to positive feedback control [for review, see Kashyap et al. (2009)]. SOX2 interacts weakly with PARP1 on regulatory elements, but upon activation of PARP1, binding between both proteins is enhanced due to auto-modification of PARP1 (Lai et al., 2012) (Figure 2B). Although SOX2 is not a direct target of PARylation, SOX2 DNA-binding is inhibited, leading to disruption of SOX2/OCT4 transcriptional complexes and induction of differentiation. Hypothetically, this is achieved by SOX2-PAR interaction, but formal proof is missing yet. This sequence of events was described in embryonic stem cells treated with retinoic acid: exposure to RA led to activation of FGF/ERK1 pathway resulting in increased PARylation of PARP1, probably by phosphorylation of PARP1, which has been shown to activate the enzyme (Kauppinen et al., 2006; Cohen-Armon, 2007). Thereafter, binding between SOX2 and PARP1 is enhanced due to auto-modification, transactivator function of SOX2 is inhibited and subsequently, differentiation of ESC is induced.

### PARP1 protein as positive co-factor in transcription

On the other hand, PARP1 is also a general activator of transcription as it is identical with positive co-factor 1 (PC1) (Meisterernst et al., 1997). Supporting this, PARP1 has been shown to associate with RNA Pol II-dependent promoters in open chromatin, whereas H1 is mainly found in heterochromatic-like regions, making their presence on chromosomes mutually exclusive (Krishnakumar et al., 2008). Specifically, E2F1 interacts with PARP1 in order to induce expression of S-phase genes such as DNA Polα/DNA primase, RPA and E2F1 itself (Simbulan-Rosenthal et al., 1999). DNA-binding or PARP1 activity is not needed for this co-activator function (Simbulan-Rosenthal et al., 2003). Similar to E2F1, another important transcription factor depends on PARP1 protein for transactivator function: NFκB, the master-regulator of immune-responsive genes (Hassa and Hottiger, 1999) (Figure 2C). PARP1 and both subunits of NFκB, p50 and p65, form a ternary complex, and without PARP1, some genes targeted by NFκB are not expressed, for example *NOS2*, coding for inducible nitric oxide synthase (Hassa et al., 2001). PARP1 activity is dispensable for co-activator function and may even inhibit NFκB-dependent transcription due to interference with its DNA binding (Chang and Alvarez-Gonzalez, 2001). There is evidence that effective NFκB-mediated transactivation of genes has several layers of regulation. PARP1 acetylation by histone acetyl-transferase (HAT) p300 is a prerequisite for binding to NFκB subunit p50, and p300 also binds and activates NFκB directly (Hassa et al., 2005). Additionally, Mediator—another co-activator complex—interacts with both NFκB and PARP1, synergistically enhancing NFκB transactivator function.

A switch between co-activating and repressive function has been described in insulin producing β-cells. At the Reg protein promoter PARP1 presence is necessary for transcription, but activation by DNA strand breaks disrupts the complex and transcription is silenced (Akiyama et al., 2001). In line, the master transcriptional regulator of genes related to energy metabolism and mitochondrial function, NRF1 (nuclear respiratory factor), is also controlled by PARP1 activity (Figure 2D). NRF1 binds PARP1 irrespective of auto-modification status, and PARP1 recruits the DNA-PK/TopoIIβ complex to NRF1-regulated promoters for expression, i.e., of the cytochrome c gene (*CYC*). As soon as NRF1 becomes a target for PARP1 activity, NRF1 loses its ability to bind PARP1 and transcription of respective genes is shut down (Hossain et al., 2009).

Thus, it seems a general feature that PARP1 functions as a nuclear sensor of stress exposure, and upon stimulation of its enzymatic activity by DNA breaks or phosphorylation, it shuts down transcription. The PARP1 protein itself may act as positive regulator for expression. In this way, a broad range of genes can be repressed that are not necessary for proper response—or even contradictory—to the imposed stress.

### PARP1 activity as positive co-factor in transcription

**Figure 3:**
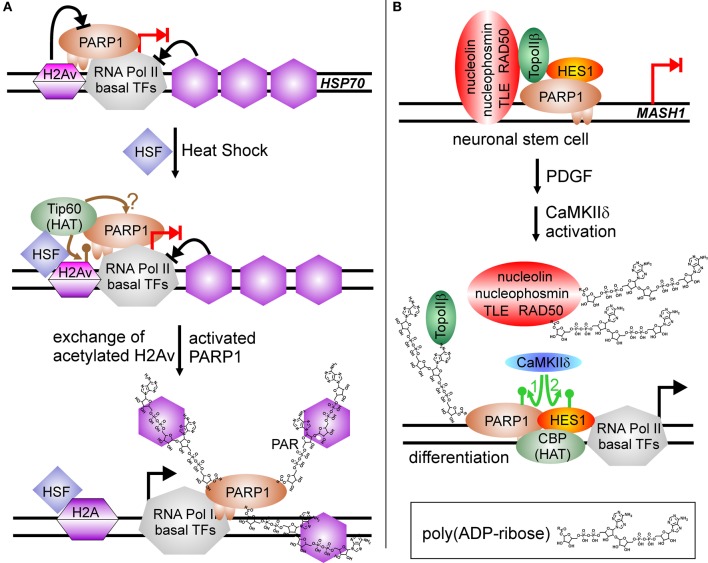
**PARP1-activity mediated stimulation of transcription. (A)** Chromatin modulation by PARP(1) activity. *HSP70* promoter is silenced by incorporated H2A variant (H2Av), blocking PARP(1) activity (blocked black arrow from H2Av to PARP1). RNA Pol II complex is assembled at promoter and poised for transcription, but specific nucleosome positioning halts RNA Pol II activity (blocked black arrow from nucleosomes to RNA Pol II). Heat shock induces the translocation of HSF (heat shock factor) to the *HSP70* promoter and recruits the histone acetyl-transferase Tip60, which induces replacement of H2Av against standard H2A by acetylation (brown lollypop). Putatively, it could also target PARP1, similar to the situation at NFκB-regulated promoters. Activated PARP1 is released from the promoter and traps suppressive histones in the growing PAR chain, facilitating transcription (switch blocked red arrow to black arrow). **(B)** PARylation activates expression of differentiation-linked genes. Treatment of neuronal stem cells with PDGF (platelet derived growth factor) induces activity of the kinase CaMKIIδ. Phosphorylation of PARP1 (green arrow No. 1) stimulates PARylation, leading to disassembly of the large co-repressor complex including nucleolin, nucleophosmin, TLE, RAD50, TopoIIβ, PARP1 and HES1 at *MASH1* promoter. Auto-modified PARP1 and HES1 recruit histone acetyl-transferase CBP, and subsequent phosphorylation of repressor protein HES1 by CaMKIIδ (green arrow No. 2) initiates transcription (switch blocked red arrow to black arrow).

However, transcriptional regulation by PARP1 grew more complicated in 2002, when a groundbreaking work appeared in *Genes and Development* and a follow up 2003 in *Science*, using *D. melanogaster* as a model (Tulin et al., 2002; Tulin and Spradling, 2003). Here, PARP1 activity is described to facilitate transcription. *D. melanogaster* encodes in its genome only two PARPs, one is similar to PARP5 (tankyrase) and the other shares substantial degree of homology with PARP1 from other organisms. In *D. melanogaster*, PARylation is needed during larval development as well as in heat shock for activation of specific genes, i.e., heat-shock protein *Hsp70*. Employing polytene chromosomes it could be visualized that hormone application or heat shock induced PARP1 activity, and that the synthesized PAR opened chromatin structure, generating so called “puffs,” which are areas of ongoing transcription. The mechanism was further elucidated by Petesch and Lis (Petesch and Lis, 2008, 2012). The heat shock factor (HSF) binds to the *Hsp70* promoter, where a stalled RNA Pol II resides, poised for transcription. HSF recruits the HAT Tip60, which acetylates histone H2A, leading to its exchange (Figure 3A). PARP1 resides dormant at the *Hsp70* promoter and its activity is rapidly induced by Tip60, either by the described histone switch or by direct acetylation. Subsequently, PARP1 modifies itself and is released from the promoter. Following this, histones are disassembled from the DNA and trapped in the growing polymer chain, paving the way for the RNA polymerase. Interestingly, mammalian cells contain the PARP1-suppressive histone macroH2A1.1 in *HSP70* genes responsive to heat shock, whereas constitutive *HSP70* promoters lack this variant (Ouararhni et al., 2006). In addition, heat shock induces expression of *HSP70* dependent on PAR synthesis, pointing to a very similar regulatory mechanism. Thus, PARP activity changes the surrounding chromatin by disengaging suppressive nucleosomal DNA binding. In the following years, this feature was extended to other factors than histones.

Similar to RA-mediated differentiation of ESC described above, PARP1 activity is involved in differentiation of neuronal stem cells, NSC, but this time as positive regulator of transcription (Ju et al., 2004) (Figure 3B). In NSC, transcription factor HES1 (Hairy/Enhancer of Split) is a negative regulator of gene expression. It interacts with the TLE (transducin-like Enhancer of split)/Groucho co-repressor complex. Groucho is able to recruit histone deacetylases, forming suppressive chromatin marks on differentiation-linked promoters like *MASH1*. PARP1 is part of this repressor complex, together with TopoIIβ, nucleophosmin, nucleolin and Rad50. Initiation of signaling events inducing differentiation by platelet-derived growth factor (PDGF) leads to activation of calcium-dependent kinase CaMKIIδ, which in turn is recruited to the *MASH1* promoter and phosphorylates PARP1. Phosphorylation activates PARP1 resulting in PARylation of co-repressor proteins, i.e., TLE/Groucho, TopoIIβ, nucleophosmin, nucleolin, Rad50, and PARP1 itself. Polymer-modified proteins except PARP1 leave the complex and histone acetylase CBP is recruited. Subsequently, HES1 is also phosphorylated by CaMKIIδ, which turns this repressive transcription factor in an activator of *MASH1* expression. Addition of a PARP1 inhibitor or a PARP1 mutant lacking polymerization activity (Glu988 to Ala988) blocked differentiation.

**Figure 4:**
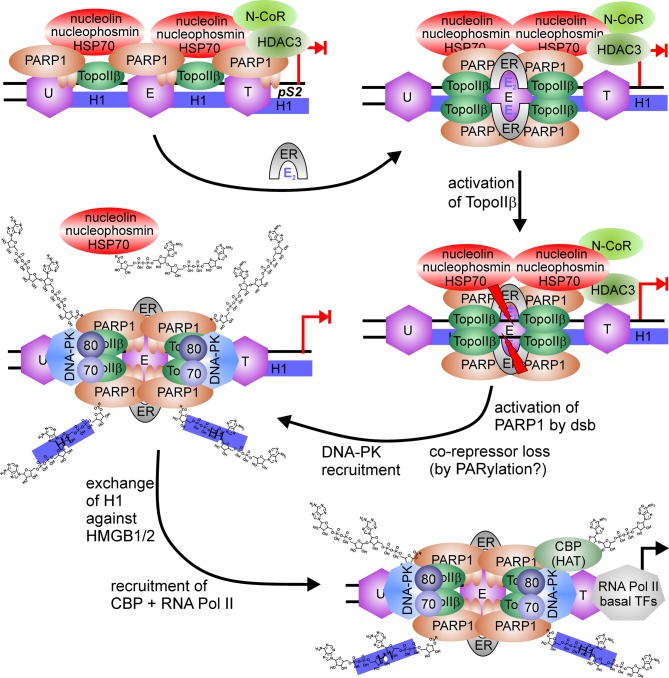
**PARP1-switch from repressor to activator of *pS2* expression.** PARP1 is member of a large co-repressor complex with nucleolin, nucleophosmin, HSP70, TopoIIβ, PARP1, and N-CoR. 17 β–estradiol (E_2_) application recruits estrogen-receptor (ER) to the ER responsive element, where the nucleosome (E) is localaized. This induces enrichment of TopoIIβ and PARP1 at the same site (E) and loss at surrounding nucleosomes located upstream (U) or downstream at the TATA-box (T). TopoIIβ induces DNA double-strand break at ERE (red flashes), which activates PARP1 and leads to the disassembly of co-repressor complex, putatively by PARylation. Additionally, DNA-PK complex (DNA-PKcs/Ku70/Ku80) is bound to the interrupted DNA strand. PARylation induces exchange of suppressive linker histone H1 against permissive HMGB1/2 protein and recruits histone acetyl-transferase (HAT) CBP.

Low levels of a similar repressor complex are found at the 17 β–estradiol (E_2_)-sensitive *pS2* promoter, composed of PARP1, TopoIIβ, nucleophosmin, nucleolin and HSP70 (Ju et al., 2006). Treatment with E_2_ leads to a rapid increase of TopoIIβ and PARP1 at the promoter, followed by recruitment of DNA-PK and co-activator CBP, whereas co-repressors are lost from *pS2* promoter (Figure 4). Formation of double-strand breaks (dsb) by TopoIIβ induces PARP1 activity and replacement of histone H1 with HMGB1/2, facilitating expression. Again, treatment with a PARP1 inhibitor or usage of the same catalytic mutant as above blocked *pS2* activation.

There are several more examples for PARP1 activity driven transcription. The repressor-activator switch has also been described in context of chromatin-modulator protein DEK (Gamble and Fisher, 2007). In a complex, DEK and PARP1 suppress transcription *in vitro* on chromatinized plasmid templates. Addition of NAD^+^ relieves suppression as both DEK and PARP1 are lost from template due to modification with poly(ADP-ribose). This enables the recruitment of the Mediator co-activator complex and subsequent transcription. PARP1 is also localized at promoters of mitochondria-related nuclear genes for DNA repair and transcription (Lapucci et al., 2011). Treatment of cells with PARP inhibitors reduces mitochondrial DNA integrity and as a consequence, expression of respiratory genes and ATP production is compromised.

Of note, PARP1 regulates its own promoter, which resembles that of TATA-less housekeeping genes. Upstream of the initiation site, there are racket-like inverted repeats, which are able to form alternative stem-loops. These structures can be bound and stabilized by PARP1, leading to abrogation of transcription. Activity of the enzyme is not necessary for repression, but would obviously release the suppression of the *PARP1* gene (Oei et al., 1994; Schweiger et al., 1995; Soldatenkov et al., 2002; Vidakovic et al., 2009). In this way, PARP1 protein keeps itself at a constant level.

### Post-translational modifications of PARP1 in transcription

In summary, PARP1 is able to regulate transcription at several levels. If PARP1 is in fact belonging to the group of general factors of RNA-PolII transcription (the missing TFIIC) may be questionable, but its interaction with several transactivator proteins is without doubt. It can act itself as a co-activator of gene expression, with the potential to abrogate transcription after activation. In this way, genes are transiently silenced that are either not needed for or may even interfere with an appropriate stress response in cells. Alternatively, PARP1 activity can rearrange nucleosomal organization and facilitate thereby accessibility of the promoter to transcription factors and RNA Pol II. In this setting, PARP1 can either be specifically recruited or may be switched from a co-repressor to a co-activator after stimulation by post-translational modification [for review, see also Kraus (2008)]. Indeed, PARP1 is targeted by many enzymatic activities. Most prominent is the auto-modification by PARylation, inhibiting DNA-binding as well as enzymatic reaction. Phosphorylation by ERK1/2 (Kauppinen et al., 2006; Cohen-Armon, 2007), AMPK (Walker et al., 2006) and CaMKIIδ (Ju et al., 2004) has been reported, stimulating PARP1. Acetylation of PARP1 also increases activity (Hassa et al., 2005), whereas SUMOylation seems to restrict protein-substrate targeting of PARP1 (Masson et al., 1997; Messner et al., 2009; Ryu et al., 2010). K48-Ubiquitination leads to degradation of PARP1 (Wang et al., 2008; Martin et al., 2009), which is probably induced by auto-modification of the enzyme (Kashima et al., 2012). Interestingly, there is crosstalk between these modifications, as SUMOylation inhibits PARP1 acetylation, thus diminishing its co-activator function in NFκB transcription (Messner et al., 2009), and for activation of the *HSP70.1* promoter in mammalian cells an ordered sequence of PARP1 modifications has been described (Martin et al., 2009): Heat shock induces activation and auto-modification of PARP1 residing at the *HSP70.1* promoter, which recruits SUMOylating enzymes Ubc9 and PIASy to this site, resulting in polySUMOylation of PARP1 and full transcriptional activation of the *HSP70.1* gene. SUMO-modification in turn attracts ubiquitin-ligase RNF4, which subsequently tags PARP1 for degradation. Promoters of inducible HSP70.1 and HSP70.2, but not of constitutive HSP70.8, are enriched of histone macroH2A1.1, which suppresses PARP1 activity. Heat shock relieves suppression (Ouararhni et al., 2006), putatively via Tip60-mediated acetylation of the histone as described in insect cells, thus facilitating PARylation reaction.

## PARP1 and CTCF in epigenetic controlling

First evidence that PARP1 plays a role in epigenetic mechanisms came from experiments utilizing PARP inhibitors. Treatment of fibroblasts with 3-aminobenzamide (3AB), a first generation PARP inhibitor with low potency, induced increased methylation of CpG islands in the *Htf9* promoter (Zardo and Caiafa, 1998), and cells displayed a rise in number and density of heterochromatic foci as well as genome-wide DNA-methylation (de Capoa et al., 1999). CCCTC-binding factor (CTCF) is known to bind regulatory regions that are hypomethylated, organizing chromatin domains as insulator and transcriptional regulator, a function which has been extensively described for the *IGF2-H19 ICR* (imprinting control region). Binding of CTCF to the non-methylated maternal *ICR*-allele facilitates *H19* transcription and silencing of *IGF2*, whereas the paternal *IGF2* gene is expressed. Loss of CTCF function increases methylation marks in respective sites and *vice versa* (CTCF is topic of several review in this special issue), i.e., in case of the *H19 ICR* not only the paternal allele, but also maternal allele is methylated. Using the *H19 ICR* as bait, CTCF was shown to be a prominent target of PARP1 activity, resulting in a molecular size shift from 130 kDa to 180 kDa (Yu et al., 2004). Covalent modification of CTCF did not interfere with its DNA-binding ability in contrast to many other proteins, but on the opposite, lack of PAR due to 3AB treatment abrogated its insulator function. Actually, CTCF bound to target sites was associated with a higher amount of PAR than free unbound CTCF.

**Figure 5:**
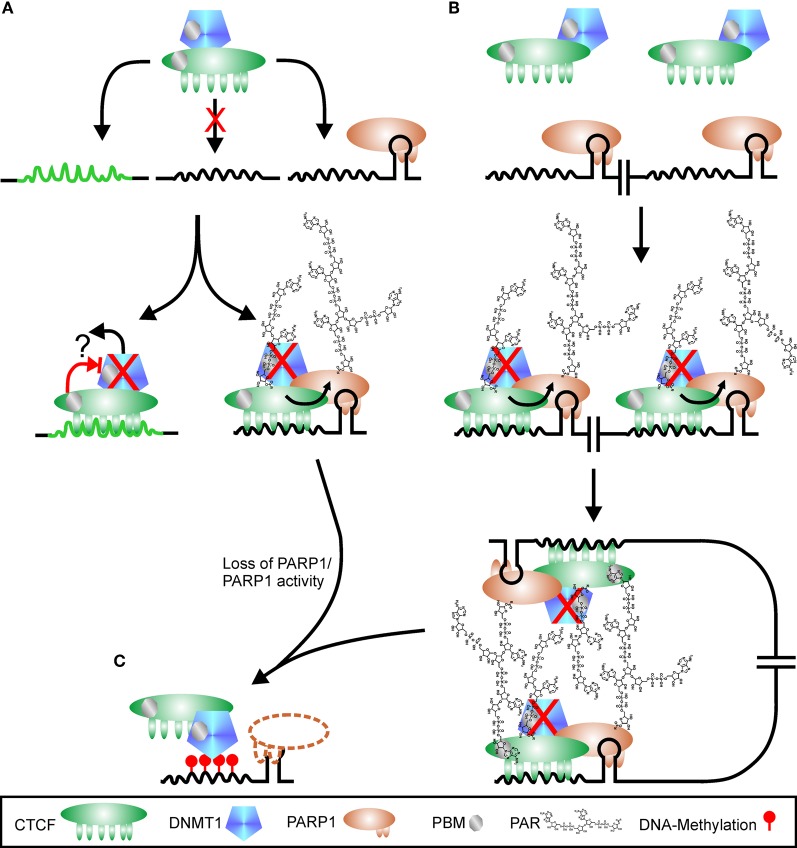
**Regulation of CTCF function by PARylation. (A)** CTCF shows a high variability in putative binding sequences. There are probably high-affinity sites (green peaked line) and low-affinity sites (black peaked line), with the latter one hypothetically only used if additional signals are present, for example PARP1 bound to a stem loop. CTCF is in complex with *DNMT1* (DNA methyl-transferase 1). Binding to high-affinity sites may suppress *DNMT1* activity directly (red cross), either by altered interaction after DNA-binding (blocked red arrow) or by release of *DNMT1* from complex (black arrow and question mark). Interaction of CTCF with PARP1 on low-affinity sites stimulates PARP1 activity, which covalently modifies itself and CTCF. *DNMT1* is inhibited (red cross) by binding to PAR via a PBM (PAR binding motif). Loss of PARP1 (dashed outline of PARP1 protein) or polymer releases suppression of *DNMT1*, and the CTCF recognition site is *de novo* methylated (red lollipops), omitting further CTCF binding **(C)**. Restructuring chromatin domains may be achieved by simultaneous usage of two adjacent CTCF-PARP1 sites as shown in **(B)**. As both CTCF and *DNMT1* contain PBMs, PAR chains may serve as “glue” between the two complexes, stabilizing chromatin loops. Loss of PARP1 or its product PAR disrupts chromatin domain organization, facilitating *DNMT1* activity **(C)**.

Soon after, another link between CTCF, PARP1 and methylation has been discovered. It was shown that DNA-methyltransferase 1 (*DNMT1*) binds to PARP1, mainly if PARP1 is auto-modified. Binding to PAR—probably via two putative PBM—inhibits DNA methylation by *DNMT1*. Interestingly, *DNMT1* has a higher affinity to PAR than to DNA, as it is case for histones (Reale et al., 2005). CTCF binds to *DNMT1* itself, but is unable to block *DNMT1* activity, so it depends on recruited PARP1 to abrogate *DNMT1* function despite physical presence. CTCF stimulates PARP1 activity even without nicked DNA, leading to an increase in PARylated PARP1 and CTCF (Guastafierro et al., 2008). In addition, the 130 kDa form CTCF was shown to bind PAR in a non-covalent manner (Figure 5) (Zampieri et al., 2012). In contrast to the negative effect on *DNMT1* activity, there is evidence that PARP1 and PARylation are needed to maintain expression of *DNMT1* in mouse L929 fibroblasts. PARP1 and PAR were detected at the *DNMT1* promoter in conjunction with *DNMT1* but without CTCF, and loss of PAR by overexpression of the degrading enzyme PARG severely reduced *DNMT1* in cells by silencing through promoter-methylation (Zampieri et al., 2009). Thus, PARP1 activity maintains transcription at the *DNMT1* promoter by keeping it clear of DNA-methylation marks inserted by *DNMT1* itself. However, an earlier publication by the same group showed the opposite effect, even in the same cell system (Zardo et al., 2002). Treatment of L929 cells with 2 mM 3AB resulted in twofold increased expression of *DNMT1*. Thus, it seems that PARP1 inhibition and increased polymer degradation by PARG overexpression may not be the same. With 3AB, PAR formation is blocked, whereas increased PARG activity induces faster loss of *synthesized* PAR. It could also be the other way round, with low-dose 3AB not preventing basal PARylation and high PARG activity leading to degradation of basal polymers. Thus, results from these two approaches may not be directly comparable.

The connection between the four players PARP1, PAR, CTCF, and *DNMT1* has been elucidated in more detail for the differentially methylated region 1 (DMR1) upstream of the *Igf2* promoter (Zampieri et al., 2012). The three proteins CTCF, PARP1, and *DNMT1* can dimerize with each other independently and form together a ternary complex, even without polymer. Most *DNMT1* is associated with CTCF, whereas only a fraction of cellular PARP1 is part of the complex. This complex binds to unmethylated CTCF target sites only. At the DMR1, all three proteins are detected, in conjunction with PAR. Overexpression of PARG leads to disruption of the complex, loss of PARP1 and CTCF and *de novo* methylation of DMR1 by the still bound *DNMT1*. The subcellular distribution of CTCF is also under control of polymer formation (Torrano et al., 2006). Differentiation of K562 myeloid cells induces translocation of CTCF from the nucleoplasm to the nucleolus, accompanied by reduction of rRNA synthesis and growth arrest. Fractionation experiments revealed that the 180 kDa (modified) form of CTCF was prevalent in nucleoli. Inhibition of PARylation by 3AB prevented relocalization of CTCF to nucleoli upon stimulus and restored nucleolar transcription. Similar results regarding control of rDNA transcription and nulceolar organization by CTCF and PARylation have been described for Drosophila (Guerrero and Maggert, 2011).

There are several examples for the impact of PARylation on CTCF function. CTCF is necessary for proper expression of tumor suppressors p16 (*CDKN2A-INK4*) and E-cadherin (*CDH*) (Witcher and Emerson, 2009) and loss of CTCF or PARP1 represses transcription of these genes. Abrogating polymer synthesis induces hypermethylation, binding of CTCF to respective regulatory sequences is lost and p16 and E-cadherin genes are silenced. In contrast, c-Myc expression was not affected by abrogating PARP1 activity. Also another tumor suppressor, p19ARF, is under control of the CTCF-PARP1-PAR complex (Farrar et al., 2010). Mutation of the potential PARylation attachment sites in CTCF led to loss of insulator function in regulation of transcription and imprinting, similar to application of a PARP inhibitor. PARP1 binds wild-type and mutant CTCF with equal efficiency, but only the wild-type version was able to maintain p19 expression, as well as proper methylation pattern at the *H19 ICR*. The authors also showed that there are genomic hot spots of interaction between CTCF and PARP1. Despite earlier suggestions, it appeared that both isoforms of CTCF, i.e., 130 kDa as well as 180 kDa, are ADP-ribosylated, but to a different extent. Whereas the larger one contains long and putatively branched polymer, the small isoform contains oligo(ADP-ribose), detected only by an antibody with high affinity to short ADP-ribose chains. As not only cell cycle inhibitors p16 and p19 are controlled by CTCF, but also c-Myc (Lobanenkov et al., 1990; Gombert and Krumm, 2009), pRb (De La Rosa-Velazquez et al., 2007), p21 and p27 (Qi et al., 2003), loss of CTCF function may support cancer formation and indeed, 87.7% of tested breast tumors showed alterations in the ratio between PARylated 180 kDa and 130 kDa forms of CTCF. Whereas normal breast tissue contains only the large isoform, both can be detected in tumor tissue. Interestingly, there is transition from CTCF-180 to CTCF-130 in primary cultures from breast tissue upon stimulation of proliferation and *vice versa*, i.e., growth arrest induces CTCF-180 (Docquier et al., 2009). This is in line with the above described observation of (Torrano et al., 2006). Despite general interaction between CTCF and PARP1 independently from other factors, CTCF function is not on all sites impaired by abrogating PARylation.

## Discussion

### PARP1 in repair

PARP1 regulating chromatin can be divided into two different major subsets: one is characterized by no or low levels of PARylation in unstimulated cells, the other by high levels of PAR as cellular stress response, but the border between these is somehow blurred. Stimulation by signaling pathways leading to phosphorylation of PARP1 at specific promoters may result in high local PARylation with no obvious change in overall polymer abundance. So, to which group does it belong? Nevertheless, massive PARylation after genotoxic stress results in changes in chromatin, which may be specific for the surrounding information or more general. Overall changes include the rearrangement of nucleosomal structure by modification of core and linker histones, which can be covalent (confined to the direct interaction with PARP1) and non-covalent, reaching beyond the proteins' localization by spreading of the PAR-“tree”. Thus, PARP1 activity clears the way for repair enzymes and complexes (see Figure 1). Additionally, the polymer is capable of attracting factors if they contain one of the three PAR-interaction modules described so far, which many proteins in DNA-maintenance pathways do. Probably, binding to polymer traps and therefore enriches respective proteins at the site of DNA breaks, and subsequent release by PARG activity enables repair of the damage. By combination of these two functions in one enzyme, chromatin loosening and protein attraction, repair rates can be accelerated. Additionally, PAR-synthesis activates the initiator kinase ATM. It has been suggested that the shift from the catalytically inactive dimer to the active monomeric form of ATM may be induced by chromatin alterations due to DNA breaks (Khanna et al., 2001), and that interaction with the MRN complex (MRE11/RAD50/NBN)—which is also a downstream target of ATM—aids in this (Assenmacher and Hopfner, 2004). The discovery of a PBM in ATM, the modulation of kinase activation by PARP inhibition and the reported direct interaction between both proteins support the hypothesis that local PAR-formation initiates the respective signaling cascade, as polymer relaxes chromatin and is bound by ATM. Thus, blocking PARP1 activity obviously slows down repair.

### PARP1 in transcription

A more specific way of mediating stress response by PARP1 activity is its participation in transcriptional regulation. Suppression of transcription in a generalized way helps to avoid additional damage induced by clash of complexes (RNA Pol II vs. DNA-repair) or possible sequence-loss caused by melting the double-strand during transcription in the vicinity of breaks. This may be facilitated by the proposed role of TFIIC/PARP1 as suppressor of nick-induced transcription via modification of basal TFs like TBP, blocking formation of PIC. But as most data supporting this came from *in vitro* experiments, this actually may be not the case in living cells. Alternatively, specific inhibition of certain promoters can be achieved in triggering PARP1 activity if the enzyme is present in the complex. Interaction with several transcription factors such as YY1, NFκB or others has been reported in several publications. Interestingly, there is mounting evidence that PARP1 acts as a switch in these complexes. For example, it is an essential co-factor of NFκB-mediated transcription, but PARylation disrupts the transcription machinery, at least *in vitro*. Similarly, polymer formation interferes with YY1 or p53 DNA binding. To complicate the whole situation, p53 displays not only three covalent attachment sites for PAR, but contains also three polymer-binding motifs. Covalent modification interferes with respective DNA binding, but strikingly abrogates nuclear export of p53 (Kanai et al., 2007); however, what is the purpose of p53 binding non-covalently to PAR? One suggestion may be the attraction and exchange of proteins at promoters. Aging and correlated oxidative stress in rat liver cells leads at the androgen receptor promoter to the exchange of positive co-factors including PARP1 against transcriptional suppressors including p53 (Shi et al., 2008). A hypothesis would be that stress-associated activation and auto-modification of PARP1 disrupts the permissive complex, and p53 is attracted by binding to synthesized polymer, resulting in silencing of the androgen receptor gene. Alternatively, retention of p53 in the nucleus may be achieved by interaction with PAR without any direct modification.

In addition, PARP1 can be activated even in the absence of DNA breaks by post-translational modifications. Phosphorylation of PARP1 mediated by CaMKIIδ after PDGF stimulation of neuronal stem cells initiates PAR synthesis at HES1-suppressed promoters. As a result, co-repressor proteins Groucho/TLE, nucleolin, nucleophosmin and TopoIIβ are released and co-activators, for example CBP, are recruited, inducing differentiation. Interestingly, PARP1 can still be found at the promoter, suggesting localization of the protein independent of its DNA-binding ability (Ju et al., 2004). If TopoIIβ activity is needed in this sequence of events has not been determined. Exchanging specific factors mediated by PARP1 activity is also seen in response to other signaling events. TopoIIβ dependent transcriptional activation is intimately associated with PARylation upon strand-break formation and subtle changes in nucleosome-positioning (Ju et al., 2006). A PARP1/TopoIIβ/DNA-PK complex is recruited to the *pS2* promoter upon stimulation of cells by estradiol and induces a DNA break. This in turn activates the PARP1 protein residing at the promoter as part of the repressor complex and modification of histone H1, which is subsequently exchanged against HMGB1, facilitating transcription. Unfortunately, the authors did not show any data about if and when proteins are PARylated. Also, the authors did not dissect the order of observed events, i.e., which is first: dsb formation by TopoIIβ or PARylation? They proposed TopoIIβ as initiating enzyme, triggering PARP1 activity, but failed to provide evidence for that. It could also well be that binding of the ER-E_2_ complex induces formation of an aberrant DNA structure by kinking the DNA, resulting in activation of PARP1. Poly(ADP-ribose) would in turn release co-repressors and H1 and recruit co-activators, i.e., DNA-PK. Subsequent dsb formation by TopoIIβ could be necessary to enable DNA binding of DNA-PK and integration of HMGB1/2 into the complex. Of note, the suppressive complex at the *pS2* promoter also contained nucleolin and nucleophosmin in addition to PARP1/TopoIIβ. Thus, these three proteins seem to be more general interacting partners of PARP1 in transcription, with nucleolin and nucleophosmin as suppressive factors, whereas PARP1 and TopoIIβ can act as switches. In addition, activity of TopoIIβ is dampened by PARP1 in mouse spermatogenesis. Inhibition of PARP1 increases double-strand break formation of TopoIIβ (Meyer-Ficca et al., 2011b), and necessary exchange of histones against protamine for compaction is disturbed, resulting in poor sperm quality and reduced fertility (Meyer-Ficca et al., 2011a). As it seems, TopoIIβ and PARP1 have a more intimate relationship in controlling chromatin and expression than thought before.

### PARP1, CTCF, and *DNMT1*

PARP activity is needed to prevent spreading of heterochromatic regions by inhibition of *DNMT1*. In addition, PARP1 interacts with chromatin-domain organizing insulator and transcription factor CTCF, which binds only to unmethylated DNA. This implies that epigenetic regulation is mediated by the interplay of PARP1, CTCF, and *DNMT1*. Lack of PAR/PARP1 or CTCF enhances the activity of *DNMT1*. Thus, the ternary complex is poised to change DNA-methylation patterns and subsequently expression profiles. Probably only basic polymer synthesis is needed for PARP1 mediated regulation of CTCF binding, as no publications are so far available that report increased CTCF localization to DNA after PARP1 activity stimulation. On the other hand, reducing PAR-levels has a dramatic impact on CTCFs DNA-binding, cellular localization and genomic methylation-pattern. If CTCF is a direct target of PARP1 or may only be recruited to PAR is still unsolved, as binding to PAR can be strong and resist general separation procedures. Alternatively, the two CTCF isoforms, i.e., 180 kDa and 130 kDa, may represent covalently modified and PAR-bound CTCF, respectively. The question is still unsolved why presence of CTCF on some genomic sites depends on poly(ADP-ribose) and on others not. Hypothetically, the high variability of CTCF binding sequences and the ability of PARP1 to bind to secondary structures may give an answer: binding of CTCF at weak interaction sites is only supported if next to the CTCF docking site a stem loop is present, bound by PARP1 (Figure 5A). Concomitant presence of the two proteins stabilizes the complex and triggers PARylation, directly stimulated by CTCF. *DNMT1* is in most cases found in association with CTCF and is therefore also recruited to the weak interaction site. Binding to the polymer abrogates *DNMT1* activity, but the enzyme is poised to methylate DNA as soon as the polymer-mark is lost (Figure 5C). At high-affinity sites, CTCF is able to bind on its own and may inhibit *DNMT1* directly or in conjunction with other proteins. Alternatively, binding of CTCF at this position may reduce affinity to *DNMT1* with subsequent loss of the methyl-transferase (Figure 5A). If two CTCF/PARP1 sites are located in close proximity due to chromatin domain organization, covalently modified CTCF can induce loop formation by interaction of its polymer-mark with the PBM of another CTCF molecule at the second position (Figure 5B), a hypothesis already raised in (Klenova and Ohlsson, 2005; Caiafa et al., 2009). It has been shown that loop-formation is one prominent feature of CTCF mediated chromatin restructuring (Yusufzai et al., 2004; Yusufzai and Felsenfeld, 2004). Auto-modified PARP1 in turn may assist in this. *DNMT1* could also be instrumental in domain formation as its own PAR-binding motif may aid in stabilizing the complex. If PARP1 or its product PAR is lost, *DNMT1* is no longer inhibited and can methylate the respective DNA sequence, abrogating CTCF binding. The hypothesis of CTCF docking sites with different affinities under putative control of PARP1 presence is supported by data presented in Witcher and Emerson (2009). Whereas the PARylation-independent CTCF-homology sequence in the *MYC* promoter displays only very weak PARP1 binding and no recruitment of TopoIIβ, PARP1 strongly interacts on its own with the PARylation-dependent *p16*/*INK4* promoter together with TopoIIβ. Alternative models have been suggested, in which CTCF is first bound to DNA and recruits in a second step PARP1 to specific sites (Caiafa and Zlatanova, 2009). CTCF-induced PARP1 activity in turn attracts *DNMT1* by binding to PAR chains. However, more recent data show that all three proteins, CTCF, PARP1, and *DNMT1*, independently interact with each other, indicating putative complex formation even in the absence of DNA (Zampieri et al., 2012). In addition, the presence of PARP1 at the silenced *p16/INK4* promoter in the absence of CTCF (Witcher and Emerson, 2009) argues in favor of the hypothesis that PARP1 independently binds to sites in the vicinity of CTCF target sequences and regulates insulator function in cases where binding of CTCF is weak.

## Concluding remarks

One major disadvantage in many newer studies tackling PARylation in transcription and chromatin organization is the use of the first-generation low-potency PARP1 inhibitor 3-aminobenzamide, and this in high doses, at which unspecific effects cannot be excluded. There are several more suitable inhibitors available such as olaparib, which has been used also in clinical trials. On the other hand, high doses of PARP inhibitors may be needed to block also unstimulated physiological PARylation. So far, no inhibitor dose-response curves have been published, analyzing especially consequences for chromatin re-organization. Adding to this, even measuring PAR levels in unchallenged cells has not been possible so far.

A yet unsolved obstacle is the experimental discrimination between covalent and non-covalent modification of proteins by poly(ADP-ribose). Addition of chaotropic agents for separation of unbound PAR from proteins may not always be successful, as in some cases interaction is strong enough to resist phenol partitioning (Panzeter et al., 1992). Non-covalent interaction can be tested by using purified PAR and recombinant proteins employing affinity assays, but the question remains if the target is also covalently modified. *In vitro* approaches to solve this problem may yield false positives, as test-tube conditions are unlikely to mirror the situation in a cell. This brings up the next question: what defines a protein respectively a specific amino acid position as substrate for PARylation? No consensus sequence has been determined yet. This leaves room for speculation, for example if only appropriate amino acids exposed in a specific 3D environment are targeted by PARP1, independent of the actual primary sequence. Recently, a MS-based method turned out to be effective in detecting covalent modification of lysines in core histone tails (Messner et al., 2010). Surprisingly, glutamates have not been found as targets for PARylation, despite earlier work defining a specific glutamic acid residue in histone H1 and in H2B as covalently modified by poly(ADP-ribose) (Ogata et al., 1980a,b). This may result from differences in the experimental approaches. Mutational analysis of potential acceptor sites in p53 strongly suggests that at least some glutamates are targeted by PARP1 (Kanai et al., 2007). Nevertheless, using MS techniques seems to be the appropriate step toward unraveling the nature of polymer target sites. In this way, also changes in phosphorylation profiles of PARP1 and PARG have been defined (Gagne et al., 2009).

Another problem arises from the combination of DNA-damage dependent stimulation and activity-related chromatin-modulating properties within one enzyme. To monitor the interaction between proteins and DNA, the method of choice is chromatin immunoprecipitation (ChIP). The sample processing includes crosslinking of proteins to DNA by administering low concentrations (about 1%) of formaldehyde to cells for a short time, usually 10 min. We proved now in a recent publication, that this procedure induces DNA strand-breaks and damage signaling itself, as detected by massive increase in PARylation and phosphorylation of H2AX (Beneke et al., 2012). This impacted on the efficiency of immunoprecipitation as suppression of both γH2AX formation and PARylation, or even PARylation alone changed the obtained results. The observed reduction in ChIP yields was specifically dependent on the monitored combination of promoter and protein. Thus, data obtained so far may be only the tip of the iceberg, as more subtle changes could be blurred by ChIP-induced DNA breaks and resulting damage signaling.

### Conflict of interest statement

The author declares that the research was conducted in the absence of any commercial or financial relationships that could be construed as a potential conflict of interest.
